# Perioperative systemic therapies for non-small-cell lung cancer: Recent advances and future perspectives

**DOI:** 10.3389/fsurg.2022.1126486

**Published:** 2023-01-20

**Authors:** Savvas Lampridis, Marco Scarci

**Affiliations:** Department of Cardiothoracic Surgery, Hammersmith Hospital, Imperial College Healthcare NHS Trust, London, United Kingdom

**Keywords:** adjuvant, immunotherapy, lung cancer, molecular targeted therapy, neoadjuvant, perioperative, surgery

## Abstract

The mainstay of treatment for early-stage non-small-cell lung cancer (NSCLC) is surgical resection. Traditionally, chemotherapy has been used perioperatively in locally extensive disease to improve the oncologic outcomes of surgery, with a 5-year absolute survival benefit of approximately 5%. In recent years, immunotherapy and molecular targeted therapy have shown excellent results in the treatment of locoregionally advanced and metastatic NSCLC, replacing chemotherapy as first-line treatment in certain cases. Consequently, researchers have been increasingly investigating the use of immunotherapy or targeted therapy in combination with surgery for the treatment of early-stage disease. This growing research interest has resulted in several published and ongoing studies of various size and design. In this mini review, we provide a succinct and up-to-date overview of recently published, phase 3 randomized clinical trials on adjuvant and neoadjuvant immunotherapy or targeted therapy for NSCLC. We subsequently discuss some important unresolved clinical issues, including the optimal duration of treatment, scheduling with respect to surgery, and potential combinations of different systemic therapies. Finally, we reference large, randomized, phase 3 studies that are currently in progress and may give answers to those and other clinical questions.

## Introduction

The standard of care for early-stage non-small-cell lung cancer (NSCLC) is surgical resection (1). Patients with stage I NSCLC who decline surgery have an estimated 5-year overall survival of as low as 11%, compared to 60%–80% in those with surgically resected disease of the same stage (2). However, patients who undergo surgery remain at substantial risk for recurrence even after complete resection of their disease. Indeed, it is estimated that 30%–75% of the patients with NSCLC who undergo surgery with curative intent develop recurrence, and they eventually die of their disease after 8–14 months (3). It therefore becomes evident that systemic anticancer therapies can be a valuable adjunct in the effort to improve the oncologic outcomes conferred by surgery.

Traditionally, chemotherapy has been the most important perioperative systemic treatment for NSCLC. The development of platinum-based combinations and the completion of randomized clinical trials assessing the activity of such regimens led to the use of chemotherapy in both the adjuvant and neoadjuvant setting. The Lung Adjuvant Cisplatin Evaluation, a pooled analysis of patient data from the five largest trials of cisplatin-based chemotherapy for completely resected stage I to III NSCLC, indicated that adjuvant chemotherapy can yield a 5-year absolute survival benefit of 5.4% (4). Similarly, a meta-analysis of individual participant data from 15 randomized controlled trials assessing neoadjuvant chemotherapy for stage IB to IIIA NSCLC showed an absolute survival improvement of 5% at 5 years, from 40% to 45% (5). Although these benefits of perioperative chemotherapy were statistically significant, there was an urgent need for enhanced treatment strategies to further improve the survival of those patients.

During the past decade, the discovery of predictive biomarkers has created new opportunities in the treatment of NSCLC. After the successful application of immune checkpoint inhibitors and molecular targeted therapies in the treatment of locoregionally advanced and metastatic NSCLC, these treatment modalities were inevitably trialed in early-stage disease in combination with surgery. As a result, there has been a recent surge of studies of various size and design in this field. The aim of this mini review is to provide a concise and up-to-date overview of recently published, phase 3 randomized clinical trials on adjuvant and neoadjuvant immunotherapy or targeted therapy for NSCLC, discuss important aspects of their application in routine practice, and identify areas for future research.

## Adjuvant immunotherapy for NSCLC

Following its successful clinical application in locoregionally advanced and metastatic NSCLC, immunotherapy has attracted growing interest for the treatment of early-stage disease. The IMpower010 trial was the first phase 3 randomized study to show significant improvement in disease-free survival with immunotherapy following adjuvant chemotherapy in patients with resected, early-stage NSCLC (6). Among 882 patients with stage II to IIIA NSCLC [as per the 7th edition of the American Joint Committee on Cancer (AJCC) staging system] who had undergone complete resection and received up to 4 cycles of adjuvant cisplatin-based chemotherapy, those randomly assigned to 16 cycles of atezolizumab experienced improvements in disease-free survival relative to best supportive care (at a median follow-up of 33 months, median disease-free survival was 42 vs. 35 months; hazard ratio, 0.79; 95% confidence interval [CI], 0.64–0.96; *P* = 0.020). A greater magnitude of benefit was observed among the 476 patients with tumors expressing programmed death-ligand 1 (PD-L1) in at least 1% of neoplastic cells (not evaluable vs. 35 months; hazard ratio, 0.66; 95% CI, 0.50–0.88; *P* = 0.004). Three-year disease-free survival rates in the overall group were 56% for atezolizumab and 49% for best supportive care, while among those with PD-L1-positive disease, the respective rates were 60% vs. 48%. Overall survival data were immature, but hazard ratio for overall survival at this early timepoint was 0.99 (95% CI, 0.73–1.33) among all patients with stage II to IIIA disease and 0.77 (95% CI, 0.51–1.17) in the subgroup of patients with PD-L1-positive tumors. The toxicity profile was consistent with that previously reported with atezolizumab monotherapy, with grade 3 or 4 adverse events occurring in 11% and grade 5 in 1% of patients, respectively. It is worth mentioning that subset analyses did not show clear benefits for atezolizumab in patients who were never-smokers, those with epidermal growth factor (EGFR)- or anaplastic lymphoma kinase (ALK)-mutated tumors, and in those with tumor expression of PD-L1 in less than 50% of neoplastic cells, although these were not powered analyses. Based on the findings of this trial, adjuvant atezolizumab is recommended in patients with completely resected, PD-L1-positive, stage II to IIIA NSCLC who received previous adjuvant platinum-doublet chemotherapy (7).

At the second prespecified interim analysis of the PEARLS/KEYNOTE-091 trial, an international phase 3 randomized study, adjuvant pembrolizumab significantly extended disease-free survival after resection of early-stage NSCLC and adjuvant chemotherapy, when indicated according to national and local guidelines (8). Among 1,177 patients with completely resected, PD-L1-positive, stage IB to IIIA NSCLC (as per the 7th edition of the AJCC staging system), adjuvant pembrolizumab improved disease-free survival compared to placebo (54 vs. 42 months; hazard ratio, 0.76; 95% CI, 0.63–0.91; *P* = 0.001), with a nonsignificant trend towards improvement in those with tumor expression of PD-L1 of 50% or more (median disease-free survival not reached in either arm; hazard ratio, 0.82; 95% CI, 0.57–1.18; *P* = 0.140). The significance boundary for overall survival in the intention-to-treat population was not crossed (18-month rate of 91.7% vs. 91.3%, respectively), but the results were immature. Grade 3 or greater adverse events occurred in 34% vs. 26% of the patients receiving pembrolizumab and placebo, respectively, without new safety signals detected. Regulatory approval prior to routine use of pembrolizumab in the adjuvant setting is awaited.

## Neoadjuvant immunotherapy for NSCLC

Similar to the adjuvant setting, immunotherapy has also been recently investigated as neoadjuvant treatment for resectable NSCLC. At the first prespecified interim analysis of the CheckMate 816 trial, an international phase 3 randomized study, among 358 patients with stage IB to IIIA NSCLC (as per the 7th edition of the AJCC staging system) and no known sensitizing EGFR mutations or ALK translocations, the addition of nivolumab to 3 cycles of neoadjuvant platinum-doublet chemotherapy significantly improved event-free survival, with a 37% reduction in the risk of disease progression, recurrence, or death, as compared to chemotherapy alone (hazard ratio, 0.63; 95% CI, 0.45–0.87; *P* = 0.005) (9). Furthermore, nivolumab improved pathological complete response rates (24.0% vs. 2.2%; odds ratio, 13.9; 99% CI, 3.5–55.8; *P* < 0.001), without decreasing the percentage of patients who underwent surgery (83.2% vs. 75.4%) or increasing the rate of grade 3 or 4 adverse events (33.5% vs. 36.9%). Although the hazard ratio for death did not cross the boundary for statistical significance, 74% of patients were still alive at the time of this analysis. Finally, treatment-related safety was consistent with that in previous reports. Based on the results of the CheckMate 816 trial, neoadjuvant nivolumab should be considered in combination with neoadjuvant platinum-doublet chemotherapy in patients with resectable NSCLC that measures 4 cm or more in greatest dimension or has regional lymph-node metastasis (7).

## Adjuvant molecular targeted therapy for NSCLC

The introduction of tyrosine kinase inhibitors in the treatment of EGFR-mutated NSCLC significantly improved the survival time of patients with locoregionally advanced and metastatic disease, and it has shown great potential in those who undergo surgical resection of early-stage NSCLC. The ADAURA trial was an international, randomized, phase 3 study assessing the role of osimertinib, a third-generation EGFR tyrosine kinase inhibitor, in completely resected, EGFR-mutated, stage IB to IIIA NSCLC (as per the 7th edition of the AJCC staging system) of non-squamous-cell histology, with or without administration of standard adjuvant chemotherapy (10). Among 682 patients, those assigned to receive osimertinib for 3 years demonstrated significantly improved 2-year disease-free survival rates compared to placebo (89% vs. 52%; hazard ratio for disease recurrence or death, 0.20; 99% CI, 0.14–0.30; *P* < 0.001). At 2 years, 98% of the patients in the osimertinib group and 85% of those in the placebo group were alive without central nervous system (CNS)-related disease (hazard ratio, 0.18; 95% CI, 0.10–0.33). The use of adjuvant osimertinib led also to a significantly reduced risk of disease recurrence or death by 83% in the subgroup of patients with stage II to IIIA NSCLC (hazard ratio, 0.17; 99.1% CI, 0.11–0.26; *P* < 0.001). The effect on overall survival remains unknown, since such data were still immature at the time of the analysis. Results of the ADAURA trial led to recommendation of adjuvant osimertinib in patients with completely resected, EGFR-mutated, stage IB to IIIA NSCLC who received previous adjuvant platinum-based chemotherapy (7).

Improvements in disease-free survival were also recently observed in 2 randomized, phase 3 studies of adjuvant gefinitib, a first-generation EGFR tyrosine kinase inhibitor, although of lesser magnitude (11, 12). In the Chinese ADJUVANT/CTONG1104 trial, adjuvant treatment with gefitinib significantly improved disease-free survival compared to chemotherapy with cisplatin and vinorelbine in patients with completely resected, EGFR-mutated, stage II to IIIA NSCLC (28.7 vs. 18.0 months; hazard ratio, 0.60; 95% CI, 0.42–0.87; *P* = 0.005) (11). Nevertheless, analysis of mature data failed to demonstrate a similar effect on overall survival. At a median follow-up of 80 months, 5-year overall survival rates with gefitinib and chemotherapy were 53.2% and 51.2%, respectively (*P* = 0.784). In the IMPACT/WJOG6410l trial, patients with completely resected, EGFR-mutated, stage II to III NSCLC who received adjuvant gefitinib experienced longer disease-free survival compared to those who received chemotherapy with cisplatin and vinorelbine (35.9 vs. 25.1 months) (12); however, the difference was not statistically significant. Interestingly, an exploratory subset analysis revealed that patients 70 years old in the gefitinib group survived longer than their counterparts in the chemotherapy group (hazard ratio, 0.31; 95% CI, 0.10–0.98; *P* = 0.046).

Icotinib, another first-generation EGFR tyrosine kinase inhibitor, was also recently assessed against platinum-based doublet chemotherapy as adjuvant treatment for completely resected, EGFR-mutated, stage II to IIIA NSCLC (as per the 7th edition of the AJCC staging system) in a Chinese, multicenter, phase 3 randomized trial (EVIDENCE) (13). At a median follow-up of 24.9 months, the median disease-free survival was significantly longer in the icotinib group compared to the chemotherapy group (47.0 vs. 22.1 months; hazard ratio, 0.36; 95% CI, 0.24–0.55; *P* < 0.001). The hazard ratio for overall survival was 0.91 (95% CI, 0.42–1.94) in the full analysis set, but overall survival data were immature. Treatment-related, serious adverse events occurred in only 1% of the patients in the icotinib group vs. 14% of those in the chemotherapy group.

## Discussion

The landscape of NSCLC treatment has changed dramatically since the advent of immunotherapy and molecular targeted therapy. In recent years, immunotherapy has shown better efficacy and lower toxicity than chemotherapy in the treatment of PD-L1-positive, metastatic NSCLC (14). Consequently, various antibodies inhibiting programmed death 1 and PD-L1 have been investigated in combination with surgery for early-stage disease. Two recent randomized, phase 3 studies confirmed longer disease-free survival with chemotherapy and immunotherapy compared to chemotherapy alone for resected, stage II to IIIA NSCLC (6, 15). In both trials, immunotherapy was administered after completion of adjuvant chemotherapy. A logical question that follows concerns the significance of the timing of immunotherapy relative to chemotherapy, as concurrent administration could be hypothesized to result in improved efficacy, but potentially increased toxicity. The answer to this question may be given by the ALCHEMIST Chemo-IO trial, an ongoing, phase 3 randomized study investigating the integration of immunotherapy to adjuvant chemotherapy for resected, stage II-IIIB NSCLC (as per the 8th edition of the AJCC staging system) (16). Recruited patients are being randomized to adjuvant platinum-based chemotherapy alone, vs. sequential chemotherapy followed by pembrolizumab, vs. concurrent chemotherapy and pembrolizumab.

Whether immunotherapy is more beneficial when administered prior to or following surgery is undetermined, and trials directly comparing the two approaches are challenging to design and conduct. Historical studies of neoadjuvant chemotherapy were underpowered, as these closed when more rapidly accruing trials of adjuvant chemotherapy demonstrated survival advantage. Nevertheless, immunotherapy may be more suitable as neoadjuvant treatment than chemotherapy, since the preoperative tumor bulk with higher levels of endogenous tumor antigen may result in presentation to, and thus priming of, more tumor-specific T lymphocytes circulating systemically (17). This systemic response continues to exert antitumor effects on the remaining neoplastic cells after surgical resection of the primary tumor, thereby potentially preventing disease recurrence (18). Another advantage of preoperative immunotherapy, as opposed to adjuvant treatment, is the assessment of tumor response in the resected specimen. Pathological response following neoadjuvant therapy in resectable NSCLC can predict survival, thus representing a prognostic factor that can inform further management strategies (19). Another significant benefit of integrating neoadjuvant immunotherapy to chemotherapy may be the radiologic downstaging of the disease, without resulting in a higher incidence or greater severity of adverse events than chemotherapy alone, and without increasing surgery-related adverse events or impeding the feasibility of surgery (9, 20). Furthermore, the addition of immunotherapy to neoadjuvant chemotherapy has been associated with more favorable surgical outcomes as compared with chemotherapy alone, with numerically shorter operating times, fewer surgery cancellations (including for disease progression), greater use of minimally invasive techniques, and fewer cases of pneumonectomy (9).

Despite the benefits of immunotherapy in the neoadjuvant setting, certain drawbacks and risks have also been noted. First, the risk of early disease progression during neoadjuvant treatment, rendering the tumor nonresectable, remains a concern. In a pilot study evaluating neoadjuvant nivolumab in resectable NSCLC, radiological reassessment with computed tomography prior to surgery did not correlate with pathological response (21). The optimal method of monitoring disease progression during or response to neoadjuvant treatment is uncertain. Second, although the toxicity of neoadjuvant immunotherapy is acceptable in results reported to date, the fact that the host immune system may be more functional in early (as compared to late) cancer stages carries the theoretical risk of marked immune-related adverse events developing concurrently with enhanced immune-mediated tumor regression (22). Finally, surgical complications as a result of neoadjuvant immunotherapy may still be a concern. Even though surgical morbidity and rates of conversion from a minimally invasive approach to open thoracotomy due to neoadjuvant immunotherapy have been reported as acceptable in multiple studies (23–25), there have also been reports of tumor-associated inflammation and fibrosis that can potentially compromise surgical plans (26).

Many other questions regarding the perioperative administration of immunotherapy for NSCLC remain unanswered, including the optimal duration of treatment, scheduling with respect to surgery, and the requirement for consolidation therapies. Ongoing and future trials will hopefully provide useful insights into these issues. Table 1 summarizes the main features of current phase 3 randomized trials investigating immunotherapy as adjuvant and neoadjuvant treatment for NSCLC.

**Table 1:** Current phase 3 randomized clinical trials of immunotherapy as adjuvant and neoadjuvant treatment for non-small-cell lung cancer.

Trial identifier (name)	NSCLC stage	Study arms	Primary endpoint
NCT02273375	IB–IIIA	Adjuvant durvalumab vs. adjuvant placebo; patients may have received prior adjuvant platinum-based chemotherapy	DFS
NCT02595944 (ALVIN)	IB–IIIA	Adjuvant nivolumab (13 cycles) following adjuvant chemotherapy vs. observation following adjuvant chemotherapy	DFS, OS
NCT03425643 (KEYNOTE-671)	II–IIIB	Neoadjuvant pembrolizumab (4 cycles) and cisplatin plus gemcitabine or pemetrexed, followed by adjuvant pembrolizumab (13 cycles) vs. neoadjuvant placebo, and cisplatin plus gemcitabine or pemetrexed, followed by adjuvant placebo	EFS, OS
NCT03456063 (IMpower030)	II–IIIB	Neoadjuvant atezolizumab (4 cycles) and platinum-based chemotherapy, followed by adjuvant atezolizumab (4 cycles) vs. neoadjuvant placebo and platinum-based chemotherapy, followed by best supportive care after surgery	EFS
NCT03800134 (AEGEAN)	II–IIIB	Neoadjuvant durvalumab and platinum-based chemotherapy vs. neoadjuvant placebo and platinum-based chemotherapy	pCR, EFS
NCT04025879	II–IIIB	Neoadjuvant nivolumab and platinum-doublet chemotherapy, followed by adjuvant nivolumab vs. neoadjuvant placebo and platinum-doublet chemotherapy, followed by adjuvant placebo	EFS
NCT04267848 (ALCHEMIST Chemo-IO)	II–IIIB	Adjuvant pembrolizumab and platinum-doublet chemotherapy (4 cycles), followed by pembrolizumab (12 or 13 cycles) vs. adjuvant platinum-doublet chemotherapy (4 cycles), followed by pembrolizumab (16 or 17 cycles) vs. adjuvant platinum-doublet chemotherapy (4 cycles), followed by observation	DFS
NCT04379635	II–IIIA	Neoadjuvant tislelizumab and cisplatin or carboplatin plus paclitaxel or pemetrexed, followed by adjuvant tislelizumab vs. neoadjuvant placebo and cisplatin or carboplatin plus paclitaxel or pemetrexed, followed by adjuvant placebo	MPR, EFS
NCT04385368 (MERMAID-1)	II–III	Adjuvant durvalumab and platinum-based chemotherapy vs. adjuvant placebo and platinum-based chemotherapy	DFS
NCT04564157 (NADIM-ADJUVANT)	IB–IIIA	Adjuvant nivolumab and carboplatin plus paclitaxel (4 cycles), followed by nivolumab (6 cycles) vs. carboplatin plus paclitaxel (4 cycles), followed by observation	DFS
NCT04642469 (MERMAID-2)	II–III	Adjuvant durvalumab vs. adjuvant placebo	DFS

DFS, disease-free survival; EFS, event-free survival; MPR, major pathological response; NSCLC, non-small-cell lung cancer; OS, overall survival; pCR, pathological complete response.

In a fashion similar to immunotherapy, molecular targeted therapy has recently occupied a prominent place in the treatment of resected NSCLC. Despite the promising results of adjuvant tyrosine kinase inhibitors, however, certain clinical questions remain unanswered. For instance, multidisciplinary tumor boards may be called to decide between adjuvant chemotherapy followed by osimertinib, as investigated in the ADAURA trial (10), or adjuvant tyrosine kinase inhibitor alone, as studied in the ADJUVANT/CTONG1104 trial (11) and the EVIDENCE trial (13). It should be argued that adjuvant cisplatin-based chemotherapy confers definite overall survival benefit and remains recommended for resected, stage II to IIIA NSCLC in the recently updated clinical practice guidelines by the American Society of Clinical Oncology (27). On the other hand, improvements in overall survival with tyrosine kinase inhibitors in the adjuvant setting has not been demonstrated thus far. Studies investigating adjuvant targeted therapy have not been powered to detect statistically significant differences in overall survival, or data on overall survival from such studies are still immature. Because its impact on overall survival is thus far unknown, patients may reasonably choose not to receive adjuvant targeted therapy.

Another question that arises from the adjuvant administration of tyrosine kinase inhibitors for NSCLC is the duration of treatment. The treatment time with osimertinib was 3 years in the ADAURA trial (10), while treatment duration was 2 years in the ADJUVANT/CTONG1104 trial (11) and in the EVAN trial, a phase 2 randomized study evaluating erlotinib vs. vinorelbine and cisplatin as adjuvant therapy in Chinese patients with EGFR-mutated, stage IIIA NSCLC (as per the 7th edition of the AJCC staging system) (28). Notably, a *post hoc* analysis of the ADJUVANT/CTONG1104 trial reported a unique spatiotemporal treatment failure pattern with adjuvant gefitinib, with cancer recurrence increasing at a steady rate 12 months following surgery and a first peak of extracranial metastases occurring 24–36 months postoperatively (29). The optimal duration of adjuvant targeted therapy remains unclear and needs additional investigation. Until then, a reasonable approach would be the administration of targeted therapy for durations used in the respective trials, with consideration also of potential toxicities of the specific tyrosine kinase inhibitor.

Neoadjuvant targeted therapies have not attracted nearly as much attention to date as have adjuvant treatments (30). The EMERGING/CTONG1103 trial has been the largest published study investigating neoadjuvant treatment with a tyrosine kinase inhibitor (31). This was a Chinese, multicenter, phase 2, randomized controlled trial comparing erlotinib with chemotherapy (cisplatin plus gemcitabine) in patients with resectable, EGFR-mutated, stage IIIA (N2) NSCLC. Improvements in the primary end point of objective response rate observed with erlotinib were not significant (54.1% vs. 34.3%; odds ratio, 2.26; 95% CI, 0.87–5.84; *P* = 0.092); nevertheless, median progression-free survival was significantly longer with erlotinib than chemotherapy (21.5 vs. 11.4 months; hazard ratio, 0.39; 95% CI, 0.23–0.67; *P* < 0.001). This advantage in progression-free survival, however, did not translate to an overall survival benefit (32). At the final analysis, after a median follow-up of 62.5 months, the median overall survival was 42.2 months in the erlotinib group and 36.9 months in the chemotherapy group (hazard ratio, 0.83; 95% CI, 0.47–1.47, *P* = 0.513). The 3- and 5-year overall survival rates were 58.6% and 40.8% with erlotinib, as compared to 55.9% and 27.6% with chemotherapy, respectively (*P* = 0.819 and *P* = 0.252 for 3- and 5-year overall survival, respectively). More randomized trials are underway, but only the NeoADAURA is a phase 3 study. This trial will evaluate neoadjuvant osimertinib with or without chemotherapy vs. chemotherapy alone in patients with resectable, EGFR-mutated, stage II-IIIB NSCLC, with major pathological response as the primary end point (33).

The relative effectiveness of different tyrosine kinase inhibitors also remains unexplored. For example, osimertinib demonstrates excellent penetrance to the CNS and has been associated with an 82% reduction in the risk of CNS disease recurrence or death in the ADAURA trial (10). In EGFR-mutated, advanced NSCLC, osimertinib showed longer progression-free survival than gefitinib or erlotinib (18.9 vs. 10.2 months; hazard ratio, 0.46; 95% CI, 0.37–0.57; *P* < 0.001) (34). Icotinib has also a lower CNS penetrance rate than osimertinib, thereby raising concerns of potential CNS recurrences (35). Future studies that will directly compare different tyrosine kinase inhibitors in the adjuvant and neoadjuvant setting will help determine which agent is more suitable for various subgroups of patients.

These and other questions may find answers in ongoing and future trials of perioperative tyrosine kinase inhibitors for NSCLC. It should be noted that some of these studies investigate targeted therapy against oncogenic driver alterations other than EGFR mutations, including ALK and ROS oncogene 1 rearrangements. Table 2 details the main characteristics of current phase 3 randomized trials of adjuvant or neoadjuvant therapy with tyrosine kinase inhibitors.

**Table 2:** Current phase 3 randomized clinical trials of tyrosine kinase inhibitors as adjuvant and neoadjuvant treatment for non-small-cell lung cancer.

Trial identifier (name)	Molecular target	NSCLC stage	Treatment strategy	Study arms	Duration of TKI	Primary endpoint
NCT01996098 (ICTAN)	EGFR	II–IIIA	Adjuvant	Icotinib for 6 months following chemotherapy vs. icotinib for 12 months following chemotherapy vs. chemotherapy	6 or 12 months	DFS
NCT02125240 (ICWIP)	EGFR	II–IIIA	Adjuvant	Icotinib vs. placebo	NA	DFS
NCT02193282 (ALCHEMIST-EGFR)	EGFR	IB–IIIA	Adjuvant	Erlotinib vs. placebo vs. observation	2 years	OS
NCT02201992 (ALCHEMIST-ALK)	ALK	IB–IIIA	Adjuvant	Crizotinib vs. observation	2 years	OS
NCT03381066	EGFR	II–IIIB	Adjuvant	Gefitinib and cisplatin plus pemetrexed (4 cycles) vs. cisplatin plus vinorelbine (4 cycles)	1 year	DFS
NCT03456076 (ALINA)	ALK	IB–IIIA	Adjuvant	Alectinib vs. platinum-based chemotherapy	2 years	DFS
NCT04351555 (NeoADAURA)	EGFR	II–IIIB	Neoadjuvant	Osimertinib vs. osimertinib and cisplatin or carboplatin plus pemetrexed (3 cycles) vs. placebo and cisplatin or carboplatin plus pemetrexed (3 cycles)	9 weeks	MPR
NCT04687241	EGFR	II–IIIB	Adjuvant	Almonertinib vs. placebo	NA	DFS
NCT04762459 (APEX)	EGFR	II–IIIA	Adjuvant	Almonertinib vs. almonertinib and cisplatin plus pemetrexed vs. cisplatin plus pemetrexed	3 years	DFS
NCT04853342 (FORWARD)	EGFR	II–IIIA	Adjuvant	Furmonertinib vs. placebo	NA	DFS

ALK, anaplastic lymphoma kinase; DFS, disease-free survival; EGFR, epidermal growth factor receptor; MPR, major pathological response; NA, not available; NSCLC, non-small-cell lung cancer; OS, overall survival; TKI, tyrosine kinase inhibitor.

In the past few years only, there has been a prosperous development of clinical trials investigating immunotherapies and molecular targeted therapies for NSCLC as adjuvant and neoadjuvant treatments. Strong evidence from phase 3 randomized studies have provided clinicians with new therapeutic options that can improve oncologic outcomes. In clinical practice, however, many questions remain unanswered and require further exploration. It is expected that current and future studies will optimize the integration of immunotherapy and targeted therapy to the perioperative patient pathway to maximize oncologic benefits and minimize treatment-related toxicities. This impending innovation represents an opportunity to improve the long-term outcomes of surgery in patients with NSCLC and ultimately change the prognosis of early-stage, potentially curable disease.
